# Pig Matrix: a matched multiomics 3D regulatory genomics database for evolutionary and comparative analyses in pigs

**DOI:** 10.1093/molbev/msag190

**Published:** 2026-07-30

**Authors:** Chao Guo, Dezhi Hua, Shuang Gan, Yue-Dong Zhang, Hang Liu, Mengting Ding, Minghao Cao, Qiuhan Wen, Chen Yan, Jing-Sheng Lu, Lei Liu, Yi-Fan Jiang, Guoqiang Yi, Zhonglin Tang, Xiang-Dong Ding, Hai-Bing Xie, Zhong-Yin Zhou, Min-Sheng Peng, Ya-Nan Wang, Xuemei Lu, Ya-Ping Zhang

**Affiliations:** School of Life Sciences, Division of Life Sciences and Medicine, University of Science and Technology of China, Hefei, Anhui 230026, China; State Key Laboratory of Genetic Evolution and Animal Models, Kunming Institute of Zoology, Chinese Academy of Sciences, Kunming, Yunnan 650201, China; Biodiversity Data Center of Kunming Institute of Zoology, Chinese Academy of Sciences, Kunming, Yunnan 650201, China; State Key Laboratory of Vegetation Structure, Functions and Construction, and Yunnan Key Laboratory of Biological Adaptation, Conservation and Utilization, School of Ecology and Environmental Science, Yunnan University, Kunming 650500, China; Southwest United Graduate School, Kunming 650092, China; State Key Laboratory of Genetic Evolution and Animal Models, Kunming Institute of Zoology, Chinese Academy of Sciences, Kunming, Yunnan 650201, China; State Key Laboratory of Genetic Evolution and Animal Models, Kunming Institute of Zoology, Chinese Academy of Sciences, Kunming, Yunnan 650201, China; Kunming College of Life Science, University of Chinese Academy of Sciences, Kunming 650204, China; State Key Laboratory of Genetic Evolution and Animal Models, Kunming Institute of Zoology, Chinese Academy of Sciences, Kunming, Yunnan 650201, China; Kunming College of Life Science, University of Chinese Academy of Sciences, Kunming 650204, China; State Key Laboratory of Genetic Evolution and Animal Models, Kunming Institute of Zoology, Chinese Academy of Sciences, Kunming, Yunnan 650201, China; Kunming College of Life Science, University of Chinese Academy of Sciences, Kunming 650204, China; State Key Laboratory of Genetic Evolution and Animal Models, Kunming Institute of Zoology, Chinese Academy of Sciences, Kunming, Yunnan 650201, China; Kunming College of Life Science, University of Chinese Academy of Sciences, Kunming 650204, China; State Key Laboratory of Genetic Evolution and Animal Models, Kunming Institute of Zoology, Chinese Academy of Sciences, Kunming, Yunnan 650201, China; Biodiversity Data Center of Kunming Institute of Zoology, Chinese Academy of Sciences, Kunming, Yunnan 650201, China; Yazhouwan National Laboratory, Sanya 572024, China; Frontier Technology Research Institute of China Agricultural University in Shenzhen, Shenzhen 518119, China; Shenzhen Branch, Guangdong Laboratory for Lingnan Modern Agriculture, Key Laboratory of Livestock and Poultry Multi-Omics of MARA, Agricultural Genomics Institute at Shenzhen, Chinese Academy of Agricultural Sciences, Shenzhen 518124, China; Shenzhen Branch, Guangdong Laboratory for Lingnan Modern Agriculture, Key Laboratory of Livestock and Poultry Multi-Omics of MARA, Agricultural Genomics Institute at Shenzhen, Chinese Academy of Agricultural Sciences, Shenzhen 518124, China; National Engineering Laboratory for Animal Breeding, Laboratory of Animal Genetics, Breeding and Reproduction, Ministry of Agriculture and Rural Affairs, College of Animal Science and Technology, China Agricultural University, Beijing 100193, China; State Key Laboratory of Genetic Evolution and Animal Models, Kunming Institute of Zoology, Chinese Academy of Sciences, Kunming, Yunnan 650201, China; Kunming College of Life Science, University of Chinese Academy of Sciences, Kunming 650204, China; State Key Laboratory of Genetic Evolution and Animal Models, Kunming Institute of Zoology, Chinese Academy of Sciences, Kunming, Yunnan 650201, China; Kunming College of Life Science, University of Chinese Academy of Sciences, Kunming 650204, China; State Key Laboratory of Genetic Evolution and Animal Models, Kunming Institute of Zoology, Chinese Academy of Sciences, Kunming, Yunnan 650201, China; Kunming College of Life Science, University of Chinese Academy of Sciences, Kunming 650204, China; State Key Laboratory of Genetic Evolution and Animal Models, Kunming Institute of Zoology, Chinese Academy of Sciences, Kunming, Yunnan 650201, China; Biodiversity Data Center of Kunming Institute of Zoology, Chinese Academy of Sciences, Kunming, Yunnan 650201, China; Kunming College of Life Science, University of Chinese Academy of Sciences, Kunming 650204, China; Yunnan Key Laboratory of Biodiversity Information, Kunming Institute of Zoology, Chinese Academy of Sciences, Kunming, Yunnan 650201, China; State Key Laboratory of Genetic Evolution and Animal Models, Kunming Institute of Zoology, Chinese Academy of Sciences, Kunming, Yunnan 650201, China; Biodiversity Data Center of Kunming Institute of Zoology, Chinese Academy of Sciences, Kunming, Yunnan 650201, China; Kunming College of Life Science, University of Chinese Academy of Sciences, Kunming 650204, China; Yunnan Key Laboratory of Biodiversity Information, Kunming Institute of Zoology, Chinese Academy of Sciences, Kunming, Yunnan 650201, China; School of Life Sciences, Division of Life Sciences and Medicine, University of Science and Technology of China, Hefei, Anhui 230026, China; State Key Laboratory of Genetic Evolution and Animal Models, Kunming Institute of Zoology, Chinese Academy of Sciences, Kunming, Yunnan 650201, China; Kunming College of Life Science, University of Chinese Academy of Sciences, Kunming 650204, China; Bio-X Center for Interdisciplinary Innovation, Yunnan University, Kunming 650500, China

**Keywords:** 3D genome, epigenome, database, distal regulation, evolution, pig

## Abstract

The pig (*Sus scrofa*) is an important model for evolutionary, comparative, and translational research; however, current functional genomic resources in pigs remain largely limited to one-dimensional genomic annotation and are therefore insufficient for systematically resolving regulatory region–gene relationships, particularly distal ones. Here, we present the Pig Matrix database, a comprehensive 3D regulatory genomics database for pigs, available at https://pigmatrix.kiz.ac.cn/. Built on a standardized experimental framework, Pig Matrix integrates matched multiomics datasets across tissues, developmental stages, and porcine cell lines, including genomic, transcriptomic, epigenomic, and 3D genome information. In total, it contains 16 library types across 7 omics layers and 7,959 processed files from 1,170 libraries. By integrating epigenomic and 3D genome information, Pig Matrix links cis-regulatory elements (CREs) to putative proximal and distal target genes, thereby facilitating interpretation of noncoding variants and genomic signals. This database provides modules for genes, candidate CREs, 3D genome architecture, genome browsing, and single-cell transcriptomics, together with dedicated evolution and comparative resources and user-oriented Genome Annotation and LiftOver tools. A representative use case illustrates how 3D regulatory annotation extends interpretation beyond linear annotation alone, recovering additional candidate genes in domestication-related signals, notably including the classical domestication gene *KIT*. Pig Matrix also incorporates xenotransplantation-related resources and may support benchmarking of AI models for regulatory genomics. Together, Pig Matrix provides an integrated platform for regulatory interpretation, evolutionary analysis, and comparative genomics in pigs.

## Introduction

The pig (*Sus scrofa*) represents an important model for investigating the genetic basis and regulatory mechanism of evolution (Groenen et al. 2012; Li et al. 2013; Ai et al. 2015; Frantz et al. 2015). Its evolutionary background is particularly informative because the expansion and dispersal history of the Eurasian wild boar (*S. scrofa*) parallels that of *Homo sapiens* in some respects: rather than through simple range expansion alone, wild boar populations expanded with admixture from related lineages, resulting in a complex demographic history (Ai et al. 2015; Frantz et al. 2015; Liu et al. 2019). This wide geographic distribution and population structure, in turn, contributed to multiple independent domestication events in domestic pigs (*S. scrofa domesticus*), which were subsequently shaped by both natural and artificial selection, leading to substantial phenotypic diversification (Larson et al. 2005, 2007; Groenen 2016; Peng et al. 2025). Meanwhile, pigs share broad anatomical, physiological, and metabolic similarities with humans, making pig–human comparative analyses valuable for understanding complex traits and disease (Yan et al. 2018; Sjöstedt et al. 2020; Lunney et al. 2021; Hou et al. 2022). With recent advances in xenotransplantation, pigs have emerged as the principal donor species, underscoring that the significance of such cross-species comparative research now extends beyond basic biology into translational medicine (Yang et al. 2015; Niu et al. 2017; Griffith et al. 2022; Anand et al. 2023; Tao et al. 2025).

Given this value, the pig is now supported by high-quality reference genomes and extensive catalogs of genetic variation (Groenen et al. 2012; Tong et al. 2023; Liu et al. 2024; Yang et al. 2024; Zong et al. 2025). However, as in humans, many trait-associated variants and genomic signals (eg GWAS hits and selection signatures) reside in noncoding regions enriched for cis-regulatory elements (CREs) (Maurano et al. 2012; Farh et al. 2015; Liu et al. 2024; Zhang et al. 2024; Xu et al. 2025). Characterizing the epigenomic landscape of these regulatory regions has become a central strategy for interpreting genome function, as demonstrated by large-scale efforts such as ENCODE and the Roadmap Epigenomics Project (Dunham et al. 2012; Kundaje et al. 2015; Moore et al. 2026). Moreover, with the development of high-throughput chromatin conformation capture technologies, the three-dimensional (3D) organization of the genome is increasingly recognized as a critical layer of gene regulation, governing how distal regulatory elements physically contact and regulate their target genes (Yang and Hansen 2024; Dekker et al. 2026).

In recent years, substantial progress has been made in functional annotation of the pig genome. Studies such as those by Zhao et al. and Pan et al. have systematically mapped large numbers of CREs across multiple tissues using linear epigenomic features, providing an important foundation for regulatory annotation (Kern et al. 2021; Pan et al. 2021; Zhao et al. 2021). However, linear annotations alone remain insufficient for resolving distal regulatory relationships. Although initial pig Hi-C studies have demonstrated the value of 3D genome mapping for identifying long-range regulatory interactions, currently available pig 3D genomic resources remain limited in tissue and developmental-stage coverage (Chen et al. 2022b; Jin et al. 2023; Lin et al. 2024; Tan et al. 2025). In addition, existing pig multiomics databases are largely built by integrating heterogeneous public datasets generated under different genetic backgrounds and experimental conditions (Fu et al. 2023; Han et al. 2025). Although these resources are highly valuable, such heterogeneity can complicate cross-omics integration and systematic regulatory interpretation. Together, studies and applications based on the pig model still lack a high-quality resource that integrates multiomics and 3D regulatory information to link genes with their regulatory elements.

To address this gap, we developed the Pig Matrix database (https://pigmatrix.kiz.ac.cn/), hereafter referred to as Pig Matrix, a comprehensive 3D regulatory database for the pig. Pig Matrix integrates matched multiomics datasets generated from a unified experimental design across diverse tissues, developmental stages, and porcine cell lines. By integrating epigenomic and 3D genome information, the database connects proximal and distal CREs to their target genes, thereby facilitating functional interpretation of noncoding variants and genomic signals in evolutionary and comparative studies. In addition to data retrieval, visualization, genome browsing, and download, Pig Matrix provides dedicated resource modules for evolutionary and comparative analyses and is further extended with xenotransplantation-related resources and potential utility for future AI-oriented applications in regulatory genomics.

## Results

### Overview of the Pig Matrix database

**Figure 1:**
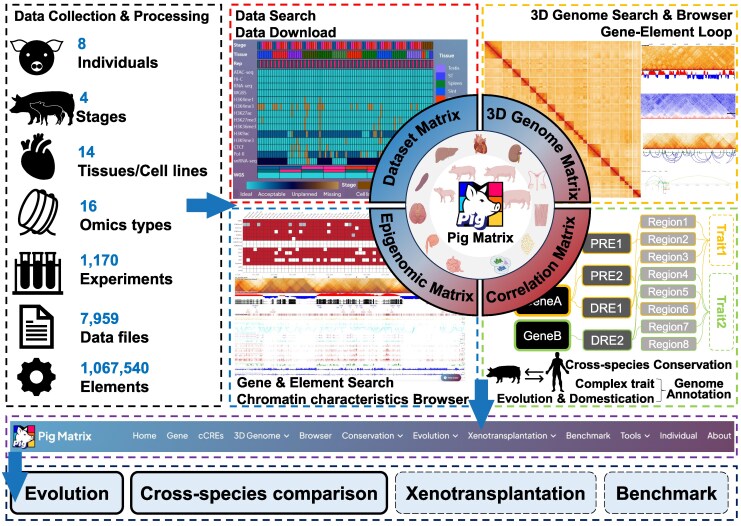
Database content and construction. Pig Matrix integrates matched multiomics and 3D genome information in pigs through four core matrices and a unified web interface, supporting data retrieval, regulatory interpretation, evolutionary and comparative analysis, as well as extended applications in xenotransplantation and AI-oriented benchmarking

Currently, Pig Matrix archives an integrated compendium of 16 multiomics library types centered around the 3D epigenome. This includes Hi-C, ATAC-seq, ChIP-seq (targeting 2 transcription factors, CTCF and Pol II, alongside 7 histone modifications: H3K4me3, H3K9ac, H3K27ac, H3K4me1, H3K36me3, H3K27me3, and H3K9me3), whole-genome bisulfite sequencing (WGBS), RNA-seq, snRNA-seq, whole-genome sequencing (WGS), and corresponding individual phenotypic data. Crucially, all of these datasets were generated from a strictly matched cohort of 94 biological samples, originating from 11 major tissues or organs across four developmental stages from eight distinct individuals, supplemented by three widely utilized porcine cell lines (PK15, ST, and PIEC). Following rigorous filtering and standardized processing, this effort culminated in 7,959 result files across 1,170 libraries, all securely hosted within Pig Matrix for free and unrestricted public use (Fig. 1).

To organize these data, Pig Matrix is structured around four core matrices: the Dataset Matrix, Epigenomic Matrix, 3D Genome Matrix, and Correlation Matrix. Rather than operating in isolation, these matrices are seamlessly interwoven to help users intuitively access and interpret the data. This integrated framework drives nine dedicated web modules, facilitating diverse functions including data retrieval, genome browsing, downstream analysis, and the functional annotation of phenotypic variation and evolution in pigs (Fig. 1).

### Data portal services

**Figure 2:**
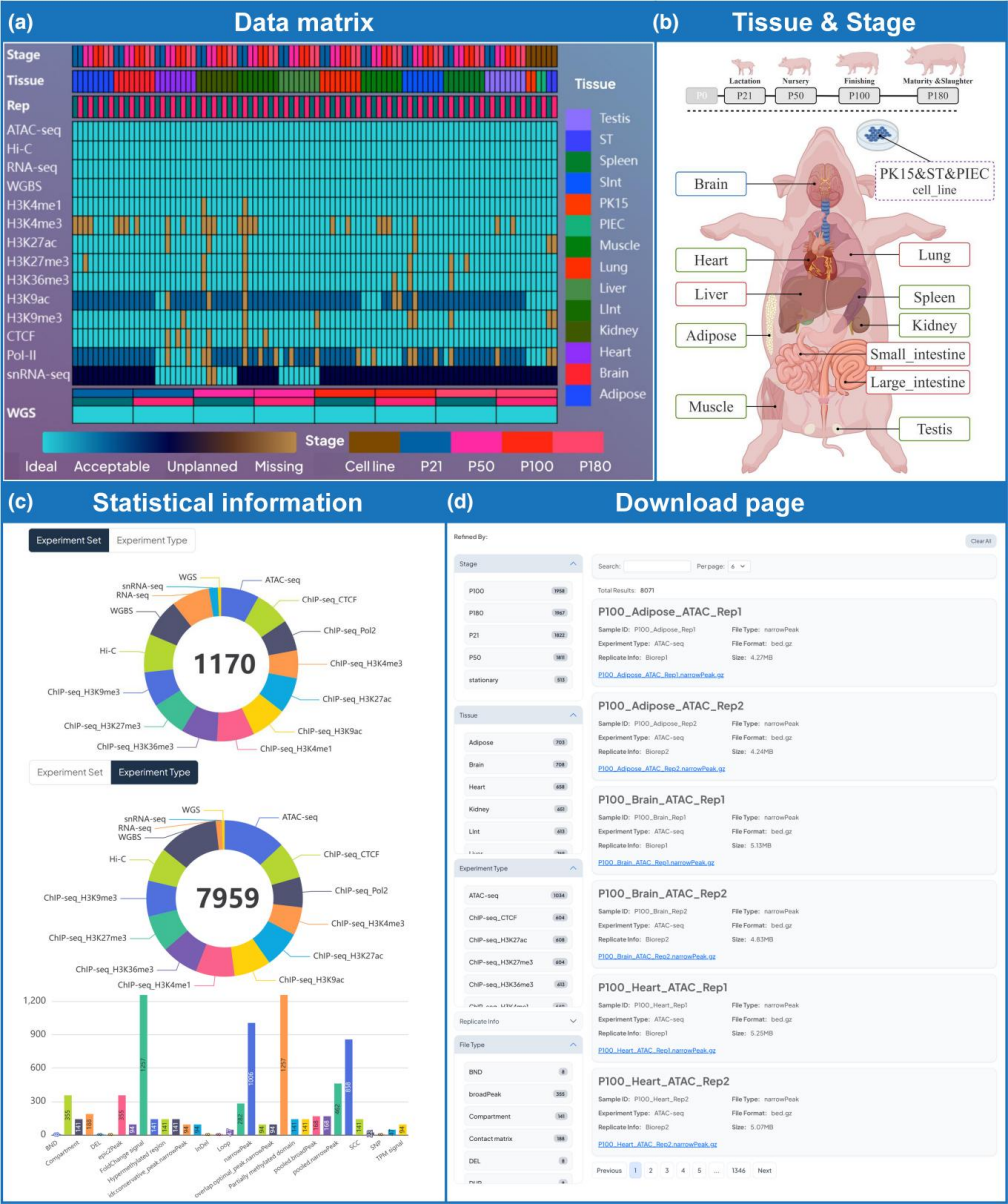
Data portal services of Pig Matrix. a) Interactive multiomics data matrix displayed on the homepage. Columns represent individual libraries organized by developmental stage, tissue, and replicate, whereas rows indicate different omics data types. Color codes denote dataset status, including ideal and acceptable released datasets, unplanned sample–assay combinations, and missing datasets excluded because of quality-control failure or sample loss. b) Anatomical and developmental schematic of the sampled tissues, organs, and cell lines represented in Pig Matrix, spanning multiple developmental stages (created with Biorender). c) Statistical summary of the database, including the total numbers of experiment sets, processed files, and their distributions across experiment types. d) The dedicated Download page, which enables users to retrieve processed files through flexible filtering by developmental stage, tissue, experiment type, replicate information, and file format.

The data portal features multiple intuitive interfaces designed to facilitate seamless data retrieval and downloading in the Pig Matrix. In the most prominent section of the homepage, we deployed an interactive multiomics matrix heatmap. This visualization provides an immediate, intuitive overview of our data scale and landscape, where distinct color blocks indicate the presence and quality metrics of corresponding omics datasets under specific spatiotemporal conditions (Fig. 2a). By clicking on a specific color block, users can filter the desired datasets and are seamlessly redirected to the download portal. The lower section of the homepage incorporates interactive anatomical and developmental schematics to represent the biological samples visually (Fig. 2b). This section provides dynamic statistics on the number of libraries and files, allowing users to perform interactive filtering directly from the diagrams (Fig. 2c). Within the Download page, which is a sub-module under the Tool menu, users can execute granular filtering by tissue, developmental stage, library type, biological replicate information, and specific file formats for unrestricted downloading data (Fig. 2d). To further increase users’ confidence in data reuse, we also added a dedicated Quality Control page under the Tool menu, where users can query library-level quality control (QC) metrics by tissue, developmental stage, library type, and keywords, with detailed explanations provided for each QC parameter.

### 3D genome module with multiscale organization of chromatin architecture

A defining hallmark of Pig Matrix, which distinguishes it from existing porcine genomic resources, is the systematic identification and cataloging of higher-order chromatin structures (Fu et al. 2023; Han et al. 2025). This module provides a comprehensive and hierarchical directory of the porcine 3D genome, organized across three scales: A/B compartments, topologically associating domains (TADs), and chromatin loops (Rowley and Corces 2018). Within this module, users can query structural elements by genomic location and sample context, and each result is annotated with key metadata such as its tissue or developmental origin and relevant quantitative scores (eg principal component analysis (PCA) scores) (Durand et al. 2016; Chakraborty et al. 2022). To ensure a transition from data retrieval to visual exploration, the location information is hyperlinked to our integrated genome browser, allowing for immediate, high-resolution visualization of the selected region. These precisely mapped 3D architectures establish the essential structural foundation for the subsequent dissection of distal gene regulation and the functional interpretation of noncoding elements.

### Gene module with proximal/distal regulation information

**Figure 3:**
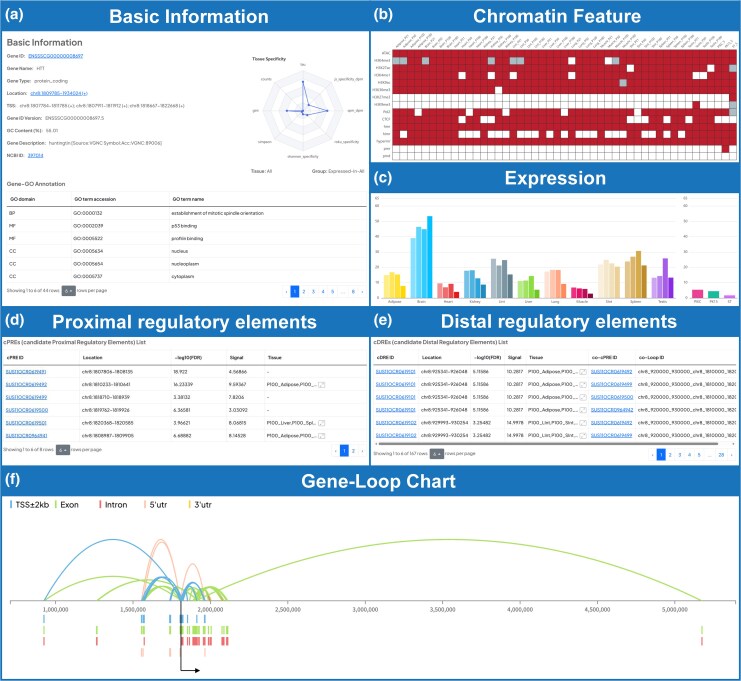
Gene-centered regulatory annotations in Pig Matrix. a) Basic Information panel of the Gene module, showing core gene metadata, genomic location, Gene Ontology (GO) annotations, and a radar plot summarizing tissue specificity across major tissues based on RNA-seq data. b) Chromatin feature panel displaying the local epigenomic landscape of the queried gene as an interactive heatmap of 15 chromatin features across spatiotemporal conditions within the promoter region (TSS ± 2 kb). Binary values indicate peak overlap status: 1 represents detected overlap with the corresponding chromatin feature, and 0 represents no detected overlap. c) Expression panel showing TPM-normalized expression levels of the queried gene across tissues, developmental stages, and porcine cell lines. d) cPREs linked to the queried gene, defined as cCREs located within the TSS ± 2 kb interval. e) cDREs linked to the queried gene through chromatin–loop interactions. f) Gene–loop chart visualizing distal chromatin interactions associated with the queried gene. Colored arcs represent chromatin loops connected to the queried gene or its associated regulatory elements. The *x* axis indicates genomic coordinates. Colored vertical marks below the *x* axis denote genomic features overlapped by loop anchors, including TSS ± 2 kb regions, exons, introns, 5′ UTRs, and 3′ UTRs. The black arrow indicates the TSS and transcriptional direction of the queried gene.

The gene module serves as a comprehensive hub for decoding the multilayered regulatory mechanisms governing individual genes. Users can precisely query target genes using gene IDs, symbols, or genomic coordinates. The basic information panel details conventional metadata, which encompasses gene biotype, transcription start site (TSS) location, and Gene Ontology (GO) terms, and is uniquely enhanced by a radar chart that quantifies tissue specificity across eight major tissues based on RNA-seq data, with the predominant tissue type formally assigned via TissueEnrich (Fig. 3a) (Jain and Tuteja 2019; Camargo et al. 2020). To show the local epigenomic landscape, the chromatin feature section employs an interactive heatmap displaying 15 distinct epigenetic marks within the promoter region (TSS ± 2 kb) across various spatiotemporal contexts (Fig. 3b). Furthermore, clustered bar charts illustrate transcripts per million (TPM)-normalized expression levels, where data from different developmental stages of the same tissue are grouped together within individual clusters (Fig. 3c).

The cornerstone of this module is the candidate CREs (cCREs) Annotation section, which is vital for parsing complex regulatory logic. Here, candidate proximal regulatory elements (cPREs) are strictly defined as cCREs located within the TSS ± 2 kb window. Conversely, candidate distal regulatory elements (cDREs) are defined as distinct cCREs that physically interact with the gene's cPREs via chromatin loops, excluding the gene's own cPREs (Fig. 3d, e).

We further contextualize these regulatory interactions within the higher-order chromatin architectures introduced previously. The module annotates the gene across three spatial dimensions: interactions are visualized using colored arc plots to intuitively map distal loop-mediated regulation (Fig. 3f); TADs catalog colocalized genes within the same topological boundary that may be subject to shared regulatory hubs; and compartments utilize a red–blue heatmap to denote transcriptionally active A and repressive B states. Finally, clustered bar charts delineate CpG DNA methylation rates across distinct gene structural components to complete the multidimensional profile.

### cCREs module with integrated multiomics information

Complementing the gene-centric perspective, the cCREs module facilitates the systematic exploration of regulatory elements, which are fully searchable by cCRE ID, tissue type, or genomic interval. The Basic Information panel outlines key identification metrics, including false discovery rate (FDR), signal intensity, evolutionary conservation, and element annotation, alongside a radar chart illustrating spatiotemporal specificity derived from chromatin accessibility (Camargo et al. 2020). Consistent with the Gene module, the chromatin feature section similarly utilizes a heatmap to map the 15 epigenomic marks across the cCRE's specific genomic region.

To elucidate the functional impact of these elements, the gene annotation section systematically reveals potential downstream targets by cataloging both proximal genes (P.Genes) and distal genes (D.Genes) physically coupled to the focal cCRE through chromatin loops.

The 3D structural context of each cCRE is similarly annotated across the three dimensions of interactions, TADs, and compartments. Notably, the interaction network uniquely records cCRE–cCRE interactions, which identify other regulatory elements connected to the target cCRE via chromatin loops. This feature empowers researchers to uncover complex higher-order regulatory logic, such as potential synergistic or competitive element–element relationships (Bell et al. 2001; Cho et al. 2018; Choi et al. 2021). Finally, clustered bar charts display the CpG DNA methylation rates of the regulatory element across different spatiotemporal conditions.

### Browser module specifically designed for the visualization of 3D epigenomics

**Figure 4:**
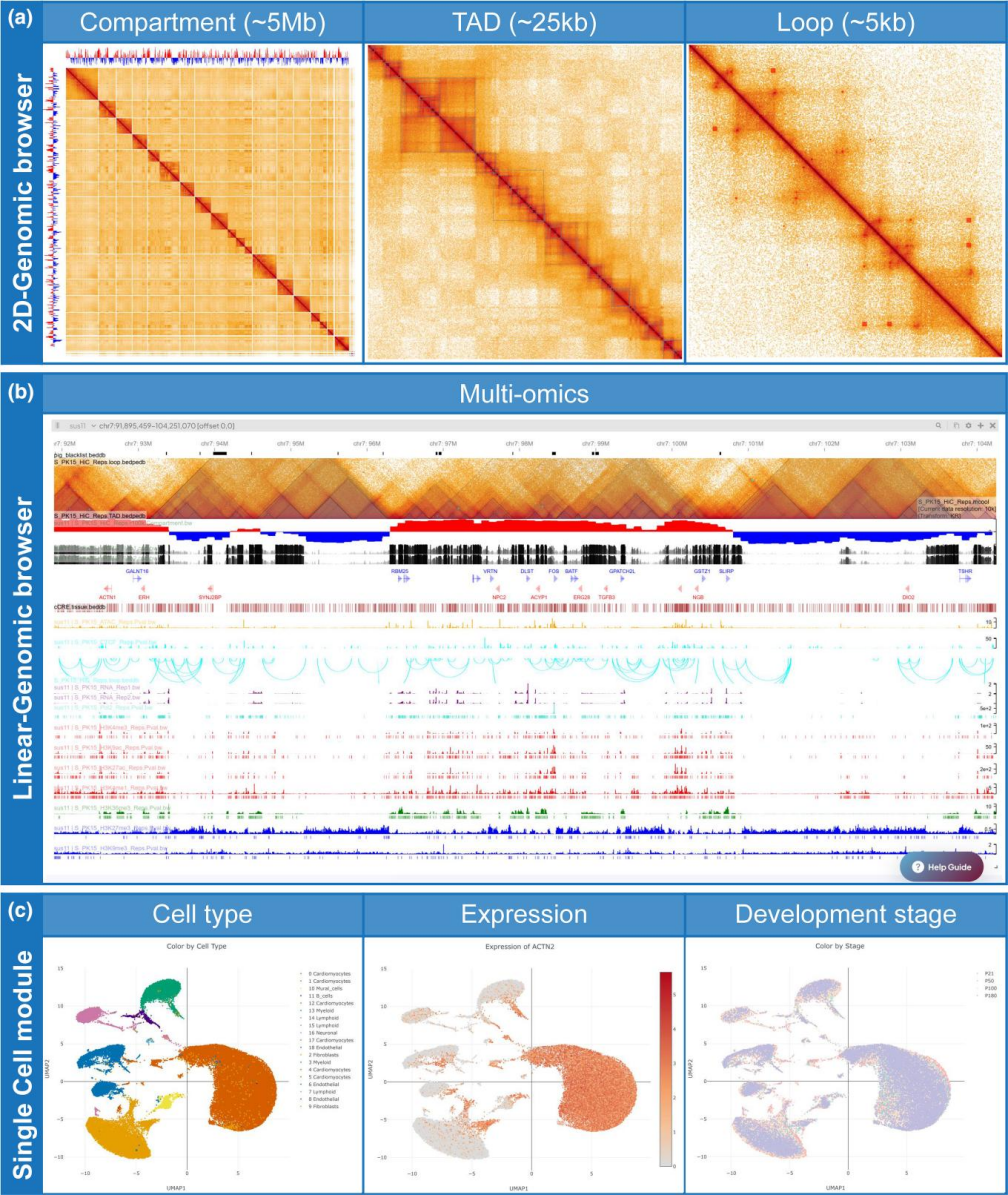
Visualization modules in Pig Matrix. a) 2D-genomic browser view displaying Hi-C contact maps at multiple structural scales, including A/B compartments, TADs, and chromatin loops. b) Rotatable linear genomic browser view enabling integrated visualization of 3D genome features together with 1D multiomics tracks. In this view, Hi-C interaction maps, TADs, chromatin loops, compartment signals, gene annotations, cCREs, and additional epigenomic tracks can be displayed simultaneously within the same genomic interval. c) Single-cell transcriptomic module for donor-relevant organs in xenotransplantation. Representative UMAP visualizations show cell-type composition, gene-expression patterns, and developmental-stage distributions in the heart, liver, and kidney across developmental stages, enabling users to examine donor-organ cellular contexts through interactive queries and metadata display.

To provide extensive support for 3D genomic data exploration, Pig Matrix integrates a customized genome browser based on HiGlass, which is a powerful framework originally developed for the massive Hi-C datasets of the 4DN project (Kerpedjiev et al. 2018; Reiff et al. 2022). This advanced browser supports the classic 2D heatmap representation of Hi-C data and features a rotatable linear heatmap view that facilitates the seamless co-visualization and integration of 3D chromatin architectures with other 1D linear multiomics tracks. Importantly, higher-order structures, including both chromatin loops and TADs, are fully rendered in both viewing modes (Fig. 4a, b). Furthermore, the browser offers extensive configuration options across various track types, empowering users to export high-quality, publication-ready vector graphics. Users can also adjust track heights and expand the display canvas using the resize handle at the bottom-right corner of the browser panel before exporting complete publication-ready figures. Finally, to assist users in mastering this platform, we have provided a dedicated tutorial that covers the entire workflow from initial data selection to final image export.

### Evolutionary resources with integrated 3D regulatory annotations

Many previous studies have identified genomic regions associated with pig domestication, adaptive evolution, and phenotypic variation, but these signals are usually dispersed across different publications and resources (Rubin et al. 2012; Bosse et al. 2014; Frantz et al. 2015; Zhu et al. 2017; Cao et al. 2025; Qiu et al. 2025; Xu et al. 2025). To facilitate their retrieval and interpretation, Pig Matrix includes a dedicated Evolution module and further annotates these regions using integrated 3D regulatory maps.

**Figure 5:**
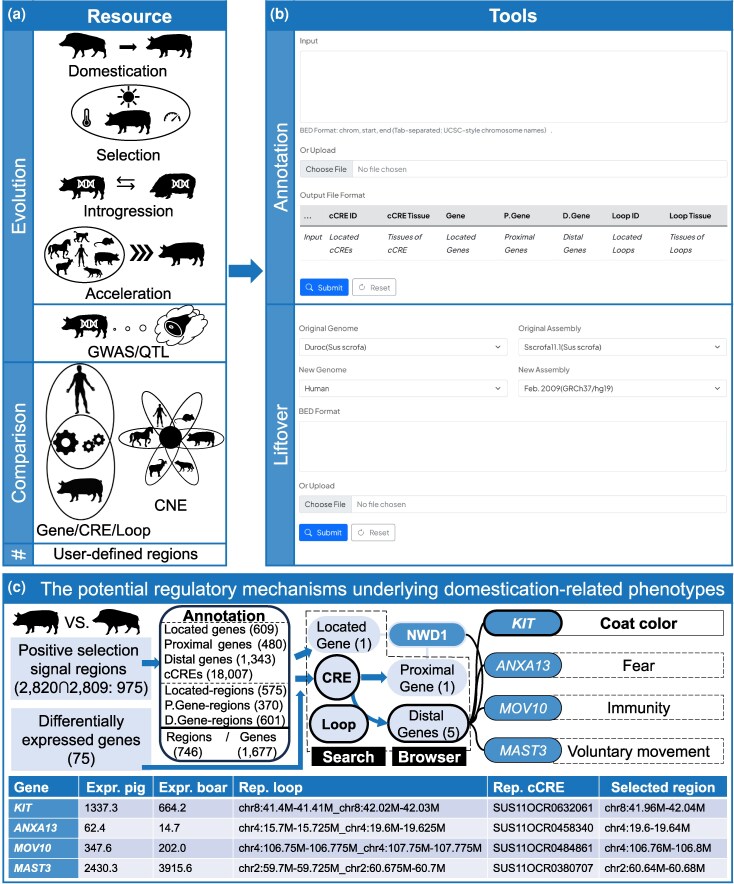
Evolutionary and comparative resources, analytical tools, and a representative use case in Pig Matrix. a) Resource modules in Pig Matrix. The Evolution module organizes genomic intervals related to domestication, selection, introgression, acceleration, and phenotype-associated GWAS/QTL signals, whereas the Comparative module integrates cross-species resources including orthologous genes, conserved CREs, conserved chromatin loops, and CNEs. b) Analytical tools implemented in Pig Matrix. The Genome Annotation tool allows users to submit genomic intervals in BED format for annotation of overlapping cCREs, located genes, proximal genes, distal genes, and chromatin loops. The LiftOver tool supports coordinate conversion between the pig reference genome and other genome assemblies for cross-breed and cross-species analyses. c) Representative use case illustrating the utility of Pig Matrix in domestication studies. Positive selection signals differentiating European domestic pigs and European wild boars were intersected with brain DEGs and annotated using the integrated regulatory framework of Pig Matrix. Compared with linear annotation alone, incorporation of 3D regulatory information recovered additional candidate genes and suggested potential regulatory mechanisms underlying domestication-related phenotypes. The implicated genes, regulatory elements, and chromatin interactions can be further explored through the Search and Browser functions of Pig Matrix. The lower table summarizes representative evidence chains linking candidate genes to putative regulatory regions, including expression levels in domestic pigs and wild boars, representative chromatin–loop interactions, representative cCREs overlapping the selected regions, and the corresponding positively selected regions.

The evolution module collects several classes of pig evolutionary and phenotype-associated genomic intervals. These include selection signatures associated with eight traits, bidirectional gene-flow regions between Asian and European pigs, domestication-related loci inferred by our group from resequencing data of nearly 1,000 individuals, lineage-specific accelerated regions (LinARs) of the Eurasian wild boar (*S. scrofa*) identified from our comparative genomic analyses, and phenotype-associated QTL and GWAS loci curated from PigQTL and PigBiobank (Hu et al. 2022; Tong et al. 2023; Zeng et al. 2024). For each interval, Pig Matrix provides annotations of overlapping cCREs, proximal and overlapping genes, distally linked genes identified through chromatin loops, and overlapping 3D genome features (Fig. 5a). In addition, these curated evolutionary and phenotype-associated intervals are preloaded as preset tracks in the HiGlass-based browser module, allowing users to visualize them together with cCREs, gene annotations, epigenomic signals, and 3D genome features within the same genomic context.

To facilitate user-defined analyses, Pig Matrix also provides Genome Annotation and LiftOver tools. The Genome Annotation tool allows users to submit genomic intervals by direct input or BED-file upload and annotates them with cCREs, located genes, proximal and distal target genes, and chromatin loops; the results can be downloaded for further exploration (Fig. 5b). In this framework, located genes are defined as genes whose bodies directly overlap an input region; P.Gene are assigned when the input region overlaps a gene-associated cPRE, defined as a cCRE within ±2 kb of the gene TSS; and D.Gene are assigned when the input region overlaps a cDRE, defined as a distal cCRE that contacts the gene-associated cPRE through chromatin–loop interactions (Fig. 5b). In addition, because Pig Matrix is anchored to the susScr11 reference genome, the LiftOver tool supports coordinate conversion between the porcine reference genome and 35 additional assemblies, including human, mouse, multiple African suid species, and numerous domestic pig breeds including several recently generated high-quality telomere-to-telomere (T2T) assemblies (eg JH, WZS, MP, and RC). These tools extend Pig Matrix from resource browsing to user-defined annotation and cross-assembly interpretation.

### A use case illustrating the utility of 3D regulatory annotation in domestication studies

To illustrate the utility of Pig Matrix for interpreting domestication-related genomic signals, we analyzed positive selection signals differentiating European domestic pigs from European wild boars. By intersecting XP-EHH (2,820) and iHS (2,809) candidates, we obtained 975 positively selected genomic regions (Table S1; Fig. 5c), which were then annotated in Pig Matrix for located genes, P.Genes, and D.Genes linked through 3D regulatory information (Tong et al. 2023). We further compared these annotated gene sets with 75 differentially expressed genes (DEGs) identified from transcriptomic comparisons between European domestic pigs and wild boars (Table S1) (Albert et al. 2012).

Overall, all 975 positively selected regions overlapped at least one cCRE, involving 18,007 unique cCREs (Table S1; Fig. 5c). Among them, 575 regions (59.0%) were associated with 609 located genes and 370 regions (37.9%) were associated with 480 P.Genes. In addition, 601 regions (61.6%) were assigned 1,343 P.Genes through 3D regulatory annotation. When located and proximal annotations were combined, 596 regions (61.1%) could be linked to 624 unique genes. By further incorporating cCRE- and loop-informed 3D regulatory annotation, the integrated framework linked 746 regions (76.5%) to 1,677 nonredundant genes (Table S1; Fig. 5c). Notably, 150 regions (15.4%) and 1,053 genes were recovered only through distal regulatory annotation, highlighting the added value of integrating cCRE annotation and chromatin-contact information for interpreting noncoding selection signals.

Intersecting these annotated genes with the 75 DEGs, only one gene, *NWD1*, was recovered through overlap- and proximity-based linear annotation (Table S1; Fig. 5c). In contrast, incorporation of 3D regulatory annotation led to the identification of four additional candidate genes, which may be relevant to morphological variation, domestication-related behavioral traits, and immune function in domestic pigs (Table S1; Fig. 5c). Among these, *KIT* is particularly notable because it is a well-established domestication-related gene underlying coat-color variation in pigs, one of the most recognizable phenotypic changes associated with domestication (Marklund et al. 1998). Importantly, *KIT* also provides a representative example showing how Pig Matrix integrates an evidence chain linking a genomic signal region (chr8:41.96M–42.04M), a regulatory element (represented by SUS11OCR0632061), chromatin architecture (represented by chr8:41.4M–41.41M_chr8:42.02M–42.03M), and a candidate target gene (*KIT*) into a single interpretable framework. This regulatory annotation can be used to further interpret the differential expression of KIT between domestic pigs and wild boars observed in the transcriptomic comparison (Fig. 5c).

*ANXA13* is also of interest, as mouse phenotype annotations (MGI: 1917037) have linked disruption of its ortholog to altered freezing behavior, which may be relevant to reduced fear and stress responses during domestication (Groza et al. 2023). *MOV10* encodes an RNA helicase involved in antiviral innate immunity and suppression of retrotransposons, providing a potential link to immune-system remodeling during pig domestication (Long et al. 2018; Nawaz et al. 2022). In addition, mouse phenotype annotations (MGI: 2683541) associate disruption of the *MAST3* ortholog with hyperactivity, classified under abnormal voluntary movement, suggesting possible relevance to behavioral adaptation from wild to captive environments (Groza et al. 2023). While *KIT* represents a well-established domestication-related gene, *ANXA13*, *MOV10*, and *MAST3* can be viewed as additional distal candidate genes that may inform future functional studies of domestication-related phenotypes. The corresponding positively selected regions, cCREs, linked genes, and chromatin interactions are listed in Table S1 and can be further explored through the Search and Browser functions of Pig Matrix. Together, this use case illustrates the unique advantage of the 3D regulatory information integrated in Pig Matrix over conventional linear annotation for interpreting domestication-related noncoding signals.

### Comparative resources for pig–human regulatory correspondence

The Comparative module provides resources for exploring pig–human regulatory correspondence across genes, CREs, chromatin loops, and conserved noncoding elements (CNEs). In addition to an orthologous-gene sub-module, it includes sub-modules for conserved cCREs, conserved chromatin loops, and CNEs. The cross pig–human cCRE sub-module is based on one-to-one orthologous relationships between porcine cCREs and the latest human cCRE catalog defined using the IPP framework, including both direct and indirect conservation types (Phan et al. 2025). The cross pig–human loop sub-module uses human chromatin–loop data from the EXPRESSO database and identifies one-to-one homologous loops according to the IPP conservation scores of their anchor regions (Cai et al. 2025). The CNE sub-module displays porcine CNEs s identified from our comparative analyses of 20 mammalian genomes and provides functional annotations for these regions (Fig. 5a). These resources provide a structured framework for comparing pig and human regulatory features and support comparative interpretation and future translational studies.

### Xenotransplantation-related resources and AI-oriented benchmarking as extended applications

Xenotransplantation represents a direct translational application of pig–human comparative biology, in which donor-pig engineering requires accurate interpretation of both coding genes and regulatory genomic contexts. To support this application, Pig Matrix incorporates a Xenotransplantation module that integrates donor-relevant genomic and transcriptomic resources (Yue et al. 2021; Anand et al. 2023; Zhu et al. 2026). The xeno-engineering genes sub-module provides a curated catalog of 24 xenotransplantation-related gene targets, including HGNC-approved gene names, human symbols, porcine ortholog symbols, pig Ensembl gene IDs, engineering strategies, functional categories, and xenotransplantation-related roles (Table S2). These genes are linked to the broader regulatory and 3D genomic framework of Pig Matrix, allowing users to examine associated cCREs, proximal and distal candidate target genes, chromatin interactions, and multiomics contexts.

Given that porcine endogenous retroviruses (PERVs) remain an important biosafety concern in pig-to-human xenotransplantation, Pig Matrix also includes a PERV TIPs sub-module that compiles PERV transposon insertion polymorphisms together with breed-of-origin information and regulatory annotations (Yang et al. 2015; Niu et al. 2017). These loci are annotated using the same framework applied throughout Pig Matrix, including overlapping cCREs, proximal and distal candidate genes, and chromatin–loop information, enabling users to evaluate whether PERV insertion sites occur within potentially functional regulatory contexts (Chen et al. 2022a). In addition, we incorporated a single-cell transcriptomic atlas of major donor-relevant organs, including the heart, liver, and kidney, across developmental stages. Through interactive uniform manifold approximation and projection (UMAP) visualizations, gene-level queries, and metadata display, users can examine cell-type composition, expression patterns, and developmental contexts in organs relevant to xenotransplantation (Fig. 4c).

In addition to these translational resources, Pig Matrix provides a benchmark-ready matched multiomics framework for AI-oriented applications in regulatory genomics (Table S3). Because chromatin accessibility, histone modifications, DNA methylation, transcription, genome variation, and 3D chromatin organization were generated or organized within a unified biological framework, Pig Matrix preserves coherent cross-modal relationships across tissues, developmental stages, and cell lines. This design can support multiple AI-oriented use scenarios, including missing-modality prediction or epigenomic imputation, regulatory readout prediction, inference of 3D chromatin features from 1D genomic or epigenomic signals, cross-species transfer beyond human- and mouse-centered training settings, and variant-aware regulatory interpretation. Together, the Xenotransplantation and AI Benchmark modules extend Pig Matrix from an evolutionary and comparative regulatory genomics database to a broader resource for translational and computational applications in pig biology.

## Discussion

Pig Matrix provides a systematically generated 3D regulatory reference for the pig by integrating matched multiomics datasets across tissues, developmental stages, and porcine cell lines. This design addresses a major limitation of many existing resources, which are often assembled from heterogeneous public datasets generated across different laboratories, platforms, and analytical pipelines, which can introduce batch effects that can complicate cross-omics integration and biological interpretation (Fu et al. 2020, 2023; Yu et al. 2024; Zheng et al. 2024; Han et al. 2025; Cai et al. 2026). By contrast, Pig Matrix integrates 3D genome information to support the assignment of candidate proximal and distal target genes to cCREs, thereby extending functional interpretation beyond linear annotation alone (Table 1) (Pan et al. 2021; Zhao et al. 2021). By integrating genome sequence, epigenomic state, chromatin architecture, and gene regulation within a single framework, Pig Matrix extends pig functional annotation toward a more interpretable platform for evolutionary and comparative analyses.

**Table 1:** Comparison of Pig Matrix with existing databases.

	Pig Matrix	PIGOME	IAnimal	ISwine
**Focus**	Pig 3D regulatory genomics	Pig functional genomics	Cross-species animal omics	Swine gene prioritization
**Species**	Pig; pig–human comparison	Pig	21 animals	Pig
**Omics**	Hi-C; ATAC-seq; WGBS; RNA-seq; snRNA-seq; WGS; ChIP-seq: CTCF, Pol II, H3K4me3, H3K9ac, H3K27ac, H3K4me1, H3K36me3, H3K27me3, H3K9me3	WGS; RNA-seq; miRNA-seq; ATAC-seq; BS-seq; MeRIP-seq; ChIP-seq: CTCF, Pol II, H3K27ac, H3K27me3, H3K36me3, H3K4me1, H3K4me3	WGS; RNA-seq; ATAC-seq; ChIP-seq: CTCF, EGR1, H3K27me3, H3K27ac, H3K4me1, H3K4me3	WGS; RNA-seq; QTX
**Data source**	Matched datasets from unified design	Public datasets	Public datasets and literature	Public datasets and literature
**Annotation**	cCREs; cPREs/cDREs; loops; TADs; proximal/distal CRE–gene links	Genes; QTLs; linear omics signals	Genes; variants; gene–trait knowledge	Variants; expression; QTX; gene ranking
**Visualization**	HiGlass-based 1D/3D genome browser	Linear genome browsers: JBrowse2, IGV	Linear genome browser and signal plots	Linear genome browser

3D genome maps provide direct physical evidence linking distal regulatory regions to candidate target genes and can support functional studies based on population-level approaches such as expression quantitative trait locus (eQTL) mapping (Chen et al. 2022a). Landmark initiatives such as GTEx in humans and the emerging FarmGTEx framework in livestock have demonstrated the value of associating genetic variation with transcriptional changes across tissues and individuals (The GTEx Consortium 2020; Teng et al. 2024). However, these approaches depend on population variation, statistical power, and sampling scale, and in domesticated species they may be further complicated by artificial selection, demographic bottlenecks, and population structure. By contrast, 3D regulatory annotation offers an orthogonal line of evidence by identifying spatially linked regulatory elements and genes, including in highly conserved regions or in genomic intervals under strong directional selection. Pig Matrix can therefore serve as a complementary resource for future pig eQTL and population-regulatory datasets.

An especially important future direction concerns xenotransplantation, one of the most direct translational applications of pig–human comparative biology. Although classical barriers remain, including immune incompatibility, coagulation and inflammatory dysregulation, physiological mismatch, and biosafety concerns such as PERVs (Peterson et al. 2024), recent kidney xenotransplantation cases indicate that the field is beginning to move beyond proof of concept toward early clinical feasibility. Notably, the living-recipient case reported by Xijing Hospital in China has been described in recent reviews as surviving for more than 8 months, with subsequent reports indicating graft function beyond 200 days (Tao and Zhou 2025). These advances make it increasingly relevant to consider a next stage of donor engineering aimed not only at overcoming major incompatibilities but also at enabling more precise and durable regulatory compatibility, including the control of cell-type-specific and distal regulatory perturbations introduced by genome editing.

A further emerging direction concerns the use of matched multiomics resources for AI-oriented regulatory genomics (Ismail et al. 2026). Recent years have seen rapid progress from sequence-based models that predict gene expression or chromatin readouts from DNA, such as Enformer, to increasingly multimodal and benchmark-driven frameworks for integrating and predicting regulatory information across omics layers (Avsec et al. 2021, 2026). However, many current models and benchmarks remain focused primarily on human and mouse data (Zhang et al. 2025). In this context, Pig Matrix provides a useful benchmark-ready framework because its genomic, transcriptomic, epigenomic, and 3D genome layers were generated from matched biological samples across tissues, developmental stages, and cell lines. This design makes the database particularly suitable for evaluating missing-modality prediction, epigenomic imputation, and the cross-species generalization of regulatory models beyond the human- and mouse-centered settings that currently dominate the field.

We will continue to develop Pig Matrix as an evolving resource for pig regulatory genomics, comparative biology, and animal-model research. A major priority will be to expand its spatiotemporal coverage by incorporating additional tissues, developmental stages, pig populations, and future data releases, with particular emphasis on embryonic and fetal stages that are likely to be critical for developmental regulation and model-related phenotypes. We also plan to integrate emerging modalities, including single-cell chromatin accessibility and single-cell 3D genome data, to improve regulatory resolution across cell types and developmental trajectories. In parallel, we will continue updating the evolutionary, comparative, and xenotransplantation-related modules as new datasets, regulatory correspondences, and donor-engineering evidence become available. Because Pig Matrix is built on a matched and extensible framework, we expect it to remain a living resource that can support not only functional interpretation of pig genomes but also long-term applications in comparative biology, translational modeling, and regulatory genomics.

## Materials and methods

### Sample collection

Pig Matrix was constructed from 94 biological samples collected from eight male Large White pigs at four developmental stages: P21, P50, P100, and P180. The pigs were collected from Chifeng City, Inner Mongolia Autonomous Region, China. Detailed individual metadata, including sample identity, developmental stage, phenotypic records, breeding information, photographs, and genetic background, are available through the Individual module of Pig Matrix. Tissue and organ samples were collected from 11 anatomical sites, including brain, heart, liver, lung, spleen, kidney, skeletal muscle, subcutaneous fat, small intestine, large intestine, and testis. After collection, samples were divided and processed according to the requirements of each omics assay and stored at −80 °C until library construction. Three established porcine cell lines were also included: PK15 and ST cells were obtained from the China Center for Type Culture Collection, whereas PIEC cells were provided by the Beijing Institute of Animal Husbandry and Veterinary Medicine, Chinese Academy of Agricultural Sciences. All cell lines were tested by MycoBlue Mycoplasma Detector (Vazyme, D101) and found to be free of mycoplasma contamination. These cell lines were maintained at 37 °C in appropriate culture media supplemented with 10% fetal bovine serum before downstream library preparation. All animal-related procedures were approved by the Institutional Animal Care and Use Committee of the Kunming Institute of Zoology, Chinese Academy of Sciences, under approval number IACUC-OE-2023-06-001.

### Library construction and sequencing

The core datasets integrated in Pig Matrix are derived from a unified experimental design covering 7 distinct omics data types: Hi-C, ATAC-seq, ChIP-seq, WGBS, RNA-seq, snRNA-seq, and WGS. For these source datasets, sequencing libraries were prepared using assay-specific protocols and sequencing platforms.

Hi-C (high-throughput chromosome conformation capture) libraries were constructed using an in situ proximity-ligation strategy with minor modifications from previously described Hi-C protocols (Lieberman-Aiden et al. 2009). Briefly, formaldehyde-fixed tissue or cell samples were lysed to release nuclei, and chromatin was digested with DpnII (NEB, R0543L). The resulting restriction-fragment overhangs were filled in using Klenow Fragment (NEB, M0210L) in the presence of Biotin-16-dCTP (Biorbyt, orb64049). Spatially proximal DNA fragments were then ligated using T4 DNA ligase (NEB M0202) under proximity-ligation conditions. After reverse crosslinking and DNA purification, ligation products were sheared by sonication to an average size of ∼300 to 500 bp. Biotin-labeled ligation products were enriched using streptavidin magnetic beads (NEB, S1420S), followed by end repair, A-tailing, adapter ligation with VAHTS Dual UMI Adapters for MGI UDB (Vazyme, NM35101), polymerase chain reaction (PCR) amplification using the *TransStart® FastPfu* DNA Polymerase kit, and purification using VAHTS DNA Clean Beads (Vazyme, N411-03). The final Hi-C libraries were sequenced on the MGI2000 platform to generate paired-end 150-bp reads.

ATAC-seq (assay for transposase-accessible chromatin with sequencing) libraries were prepared to profile genome-wide chromatin accessibility using TruePrep DNA Library Prep Kit V2 for Illumina (Vazyme, TD501). Briefly, nuclei were isolated from tissue or cell samples and subjected to Tn5 transposase-mediated fragmentation and adapter insertion. For representative reactions, ∼50,000 nuclei or cells were used as input. The transposition reaction was performed at 37 °C, and the resulting DNA fragments were purified using ZYMO DNA Clean & Concentrator-5 (Zymo, D4014). ATAC-seq libraries were amplified using indexed primers supplied with the library-preparation kit and purified using VAHTS DNA Clean Beads (Vazyme, N411-03). Final libraries were quantified using a Qubit fluorometer, assessed for fragment-size distribution using an Agilent 2100 Bioanalyzer and sequenced on the Illumina NovaSeq 6000 platform to generate paired-end 150-bp reads.

ChIP-seq (chromatin immunoprecipitation sequencing) libraries were generated to profile transcription-factor binding and histone-modification landscapes, following a previously described protocol with minor modifications (Wu et al. 2022). Formaldehyde-crosslinked tissue or cell samples were lysed using cell lysis buffer and SDS lysis buffer supplemented with Protease Inhibitor Cocktail (MCE, HY-K0010). Chromatin was sheared by sonication, and the soluble chromatin fraction was incubated with target-specific antibodies against including H3K27me3 (Diagenode, C15410069-50UG), H3K27ac (Diagenode, C15410174-50UG), H3K4me1 (Diagenode, C15410037), H3K4me3 (Diagenode, C15200152), H3K36me3 (Diagenode, C15210013), H3K9me3 (Diagenode, C15410193), H3K9ac (CST, 9649S), CTCF (Diagenode, C15410210), and Pol II (CST, 2629S). Antibody-bound chromatin was captured using Protein A/G Magnetic Beads (MCE, HY-K0202), followed by sequential washes with low-salt, high-salt, LiCl, and TE buffers. After elution and reverse crosslinking, ChIP DNA was purified using Zymo ChIP DNA Clean & Concentrator (Zymo, D5205). Sequencing libraries were constructed using VAHTS Universal Pro DNA Library Prep Kit for Illumina (Vazyme, ND608-01) and VAHTS Dual UMI UDI Adapters Set1–Set4 for Illumina (Vazyme, N351), followed by PCR amplification and purification using VAHTS DNA Clean Beads (Vazyme, N411-03). The final ChIP-seq libraries were sequenced on the Illumina NovaSeq 6000 platform to generate paired-end 150-bp reads.

WGBS libraries were constructed to generate genome-wide DNA methylation profiles. Genomic DNA was extracted from tissue samples using the FastPure Cell/Tissue DNA Isolation Mini Kit (Vazyme, DC102-01) according to the manufacturer's instructions. Briefly, 50 to 100 mg minced or ground tissue was lysed and digested with Proteinase K, followed by column-based DNA purification and DNA elution. DNA integrity was assessed by agarose gel electrophoresis, and DNA concentration and purity were evaluated using a NanoDrop spectrophotometer. WGBS libraries were prepared using MGIEasy Whole Genome Bisulfite Sequencing Library Prep Kit (BGI-Shenzhen, China). Genomic DNA was mixed with 1% unmethylated lambda phage DNA as a bisulfite-conversion control and fragmented using a Covaris LE220 focused ultrasonicator. Fragmented DNA was size-selected, end-repaired, A-tailed, and adapter-ligated. Adapter-ligated DNA was bisulfite-converted using EZ DNA Methylation-Gold Kit (Zymo Research, D5006), followed by PCR amplification and library quality control. The final libraries were sequenced on the MGI2000 platform to generate paired-end 100-bp reads.

RNA-seq (bulk RNA sequencing) libraries were constructed to quantify bulk gene-expression profiles. Tissue samples were preserved in RNAlater (Invitrogen, AM7024), and total RNA was extracted from tissue or cell samples and subjected to quality control before library construction. RNA-seq library construction and sequencing were performed by Novogene Co., Ltd. (Beijing, China), using a standard rRNA-depleted, strand-specific RNA-seq workflow. Briefly, ribosomal RNA was depleted from total RNA using an rRNA-specific probe-based strategy, and strand-specific libraries were prepared through RNA fragmentation, cDNA synthesis, dUTP-mediated second-strand synthesis, end repair, A-tailing, adapter ligation, fragment selection, and PCR enrichment. Final libraries were quality-controlled, pooled, and sequenced on the Illumina NovaSeq 6000 platform to generate paired-end 150-bp reads.

The snRNA-seq (single-nucleus RNA sequencing) libraries were constructed to profile single-cell transcriptomes at the single-nucleus level. Briefly, tissue samples were homogenized and lysed under nuclei-preserving conditions to release nuclei. The nuclei suspension was filtered to remove tissue debris and aggregates, followed by nuclei counting and quality assessment. Purified nuclei were loaded onto the MobiCube platform (MobiNova-100) for droplet generation, nucleus capture, barcoding, and reverse transcription using MobiCube high-throughput single-cell 3′ transcriptome reagents v2.1 (PN-S050400301). After reverse transcription, emulsions were broken, reverse-transcription products were purified, and cDNA was preamplified. Amplified cDNA was purified using SPRIselect beads (Beckman Coulter, B23317), quantified using Qubit 1× dsDNA HS Assay Kit (Invitrogen, Q33230), and assessed using an Agilent 2100 Bioanalyzer with Agilent High Sensitivity DNA Kit (Agilent, 5067-4626). Sequencing libraries were then generated through cDNA fragmentation, adapter ligation, index amplification using the MobiCube 3′ Dual Index kit, and final size selection. The resulting snRNA-seq libraries were sequenced on the Illumina NovaSeq 6000 platform to generate paired-end 150-bp reads.

WGS libraries were constructed from genomic DNA extracted from donor individuals for variant discovery and individual-level genetic background characterization. Genomic DNA was extracted using the same DNA extraction strategy described for WGBS, and WGS libraries were prepared using BGI Optimal DNA Library Prep Kit (BGI-Shenzhen, China). Briefly, genomic DNA was fragmented, size-selected using magnetic beads, end-repaired, A-tailed, adapter-ligated, PCR-amplified, and subjected to library quality control. The final libraries were sequenced on the MGI2000 platform to generate paired-end 150-bp reads.

Together, this library-construction framework yielded 1,170 libraries across 16 library types, from which sequencing reads were generated for subsequent standardized data processing and database integration.

### Data collection and standardized processing pipelines

Pig Matrix integrates seven distinct omics data types, all generated through a unified experimental design and analyzed via standardized, state-of-the-art pipelines.

#### 3D genome

For Hi-C datasets, upstream processing, which encompasses alignment, filtering, matrix aggregation, and normalization, was conducted in accordance with the analytical framework of the Hi-C Processing Pipeline (version 1.1, https://data.4dnucleome.org/resources/data-analysis/hi_c-processing-pipeline) of 4DN project. Subsequently, multiscale structural features were called utilizing JuicerTools (version 1.22.01), dcHiC (version 2.1), and FitHiC2 (version 2.0.8) (Durand et al. 2016; Kaul et al. 2020; Chakraborty et al. 2022; Reiff et al. 2022).

#### Epigenome

For ATAC-seq, ChIP-seq, and WGBS datasets, the official ENCODE project pipelines (https://github.com/ENCODE-DCC) were deployed locally via singularity (version 3.8.6). For ATAC-seq data, the complete analysis workflow was performed using the latest ENCODE-DCC/atac-seq-pipeline (version 2.2.2). Because the standard ENCODE-DCC/chip-seq-pipeline2 (version 2.2.2) lacks support for broad peak calling, we specifically employed epic2 (version 0.0.52) to identify broad peaks for the H3K27me3, H3K4me1, and H3K36me3 histone modifications. For WGBS data, CpG methylation rates were quantified using the ENCODE-DCC/wgbs-pipeline (version 1.1.8), followed by the calling of five distinct genomic methylation features via DNMTools (version 1.4.4, https://dnmtools.readthedocs.io/en/latest) (Stovner and Sætrom 2019; Hitz et al. 2023).

#### Transcriptome

Bulk RNA-seq data were aligned and quantified using HISAT2 (version 2.2.1) and StringTie (version 2.1.2) (Pertea et al. 2015; Kim et al. 2019). For single-nucleus RNA-seq (snRNA-seq) datasets, FASTQ files were first processed into gene-expression matrices using MobiVision (version 3.2, https://www.mobidrop.com/cn/bioinformatics-analysis-software/mobivision-news/software-download), followed by clustering and cell-type annotation utilizing Seurat (version 4.1.4) (Hao et al. 2021).

#### Genome

WGS data were processed following the GATK4 (version 4.1.3) Best Practices Workflows for alignment, as well as SNP and InDel calling and filtering (Van der Auwera et al. 2013). Structural variants were subsequently detected using smoove (version 0.2.8, https://github.com/brentp/smoove).

Ultimately, this systematic analytical framework yielded a total of 7,959 processed files, comprehensively capturing the multidimensional biological information embedded within these multiomics layers (Table 2). All processed datasets are securely hosted within Pig Matrix and are freely available for unrestricted download via a dedicated data portal.

**Table 2:** Data collection and preprocessing.

Library type		Upstream-processing tools	Feature analysis tools	Processed file information	Processed file type	Experiment number	File number
Hi-C		4DN/Hi-C processing pipeline (bwa-mem; pairtools; cooler; juicer)	JuicerTools; dcHiC; FitHiC2	interaction matrix; compartment; TAD; Loop; significant chromatin contact (SCC)	hic; mcool; cool; BigWig; Bedpe	94	564
ATAC-seq		ENCODE-DCC/atac-seq-pipeline;	macs2	signal; narrow peak	BigWig; Bed	94	1,034
ChIP-seq	CTCF	ENCODE-DCC/chip-seqa-pipeline2	macs2	signal; narrow peak	BigWig; Bed	88	604
	Pol II					76	500
	H3K4me3					69	461
	H3K27ac					88	608
	H3K9ac					88	608
	H3K4me1		epic2	signal; broad peak	BigWig; Bed	92	640
	H3K36me3					89	613
	H3K27me3					88	604
	H3K9me3					86	590
WGBS		ENCODE-DCC/wgbs-pipeline	DNMTools	methylation rate; hypomethylated region (hmr); highly methylated region (himr); hypermethylated region (hypermr); partially methylated region (pmr); partially methylated domain (pmd)	BigWig; txt; Bed	94	987
RNA-seq		Hisat2	stringtie	signal; gene expression	BigWig; tsv	94	95
snRNA-seq		MobiVision	Seurat	gene expression; cell-type annotation	seurat.rds	22	3
WGS		bwa-mem	GATK; smoove	SNP; Indel; SV	vcf	8	48

### Identification of cCREs

The identification of porcine cCREs was adapted from the representative DNase hypersensitivity sites calling strategy defined in the latest ENCODE cCRE pipeline (https://github.com/weng-lab/ENCODE-cCREs/tree/master/Version-4/cCRE-Pipeline) (Moore et al. 2026). To comprehensively capture potential open chromatin regions (OCRs) across the pig genome, we integrated our 94 newly generated ATAC-seq datasets with two large-scale public porcine ATAC-seq cohorts, utilizing these as a robust alternative to ENCODE's traditional DNase-seq approach (Kern et al. 2021; Zhao et al. 2021). To ensure the high fidelity and robustness of the resultant representative OCRs (rOCRs), raw ATAC-seq peaks were subjected to a stringent four-step filtering regimen: (i) applying an initial significance threshold of FDR < 0.001; (ii) excluding peaks overlapping with known genomic blacklist regions; (iii) retaining only peaks within the top 90% of fold-change signal intensity across all samples; and (iv) requiring strict overlap with conservative narrow peaks (cOCRs) derived from biological or pseudo-replicates. This final reproducibility criterion was specifically implemented to compensate for the current absence of a published consensus OCR catalog for the pig. Ultimately, the rigorously filtered set of rOCRs was formally designated as the final catalog of cCREs in the Pig Matrix.

### Comparative genomic analysis

For comparative genomic analyses, we used two species sets: 22 mammalian genomes (including 2 outgroups) for the mammal-wide analysis, and 33 suid genomes (including 3 outgroups) for the Suidae-wide analysis (Table S4). Whole-genome alignments were conducted across species using Cactus (version 2.7.0) (Paten et al. 2011). The MAF files were generated with GRCh38 and susScr11 genomes as the reference, utilizing haltools (version 2.2) with the flags “-onlyOrthologs -noAncestors -noDupes” (Hickey et al. 2013).

The multiple alignments were filtered to ensure that at least 90% of the species were included in the whole-genome alignments. Evolutionarily conserved elements were predicted using the PHAST package (version 1.6) (Hubisz et al. 2011). A phylogenetic tree model was built with phyloFit from the PHAST package, incorporating evolutionary distances at neutrally evolving sites and a substitution rate matrix. The model used the general reversible substitution model derived from 4-fold degenerate sites. Highly conserved elements (HCEs) were identified with PhastCons using the options -most-conserved -score. These HCEs were then classified into coding and noncoding regions based on the human and pig reference genome annotations.

We identified LinARs within the HCEs in the whole-genome alignments. The phyloP program from PHAST was used to estimate acceleration scores with the parameters -method LRT, -branch, -mode ACC for selected subgroups. The acceleration scores were adjusted for multiple testing using the FDR method. Acceleration with FDR-adjusted *P*-values ≤ 0.05 was considered significant and included in subsequent analyses.

### Database implementation

The Pig Matrix platform features a four-tier architecture, comprising an external data processing layer—utilizing C# and Python (python.org) scripts for ETL data and file ingestion—and a core web system deployed within a CentOS Linux server. Within the server, Nginx acts as a reverse proxy managing SSL, static assets, and incoming HTTP requests, routing them to the backend & logical layer built upon the ASP.NET Core application framework (dotnet.microsoft.com). This backend integrates Razor for server-side rendering, MVC Controllers, RESTful APIs, and an integrated HiGlass Service, leveraging Dapper (learndapper.com) as the data access layer (ORM) for data retrieval. The data storage layer employs a hybrid strategy: SQL Server 2019 for relational database management, MinIO (min.io) for object storage, and a dedicated file system for visualization data (labeled as HiGlass data files). Finally, the browser-based frontend layer combines foundational UI elements (HTML, CSS, JavaScript, jQuery, Bootstrap) with advanced visualization libraries—Plotly.js (plotly.com) for single-cell UMAP visualizations, EChart.js (echarts.apache.org) for diverse statistical plots, D3.js (d3js.org) for rendering gene loop diagrams, and Higlass.js (higlass.io) for interactive 3D genome tracks—asynchronously retrieving data via HTTP responses to deliver a seamless and highly responsive user experience (Figure S1) (Kerpedjiev et al. 2018).

### Tools integration and deployment

To build a robust and scalable workflow for data analysis, the Pig Matrix web server integrates two core analytical tools: LiftOver and Genomic Annotation.

#### LiftOver module

To ensure stable input/output streams and parameter settings, the LiftOver backend utilizes a modular design and containerized architecture. The core conversion engine is deployed on a Linux computing server using Docker, integrating the widely used UCSC LiftOver software (genome.ucsc.edu). The web interface allows users to input standard BED files or text. Through open APIs, the backend dynamically matches the corresponding index chain files to perform precise genomic coordinate conversions. Notably, in addition to supporting bidirectional conversions between the pig reference genome (susScr11) and human (hg19/hg38) or mouse (mm10/mm39) assemblies, this module provides extensive support for conversions between susScr11 and over 30 distinct pig breeds and related suid assemblies (eg Bama Xiang, Meishan, and European wild boar).

#### Genomic annotation module

To achieve high-performance biological feature annotation without overloading server memory, this module was natively developed using the ASP.NET Core MVC framework and deployed via a RESTful API. The backend preloads multiple high-quality susScr11-based genomic interval datasets—including genes (CDS/exons), cCREs, proximal/distal regulatory networks (cPREs/cDREs), and 3D chromatin loops (BEDPE format). To optimize querying speed, we implemented a custom fixed-size binning algorithm (bin size = 10 kb) that indexes these massive genomic intervals in server memory, avoiding I/O bottlenecks. Furthermore, the API employs asynchronous stream processing to handle large user-uploaded BED files (up to 60 MB). The annotated results are dynamically written to temporary files and automatically uploaded to a MinIO object storage server, which instantaneously generates a public download link for the user, ensuring a highly concurrent and cloud-native user experience.

## Supplementary Material

msag190_Supplementary_Data

## Data Availability

The data underlying this article are available in Pig Matrix at https://pigmatrix.kiz.ac.cn/.
