# Supplementary material for: Pig Matrix: a matched multiomics 3D regulatory genomics database for evolutionary and comparative analyses in pigs
Source: Mol Biol Evol. 2026 Jul 30;43(8):msag190. doi: 10.1093/molbev/msag190 (PMC13455638; doi:10.1093/molbev/msag190)
Supplement: Supplementary file 1 [file msag190_supplementary_data.zip › Figure S1 (with legend).PDF]

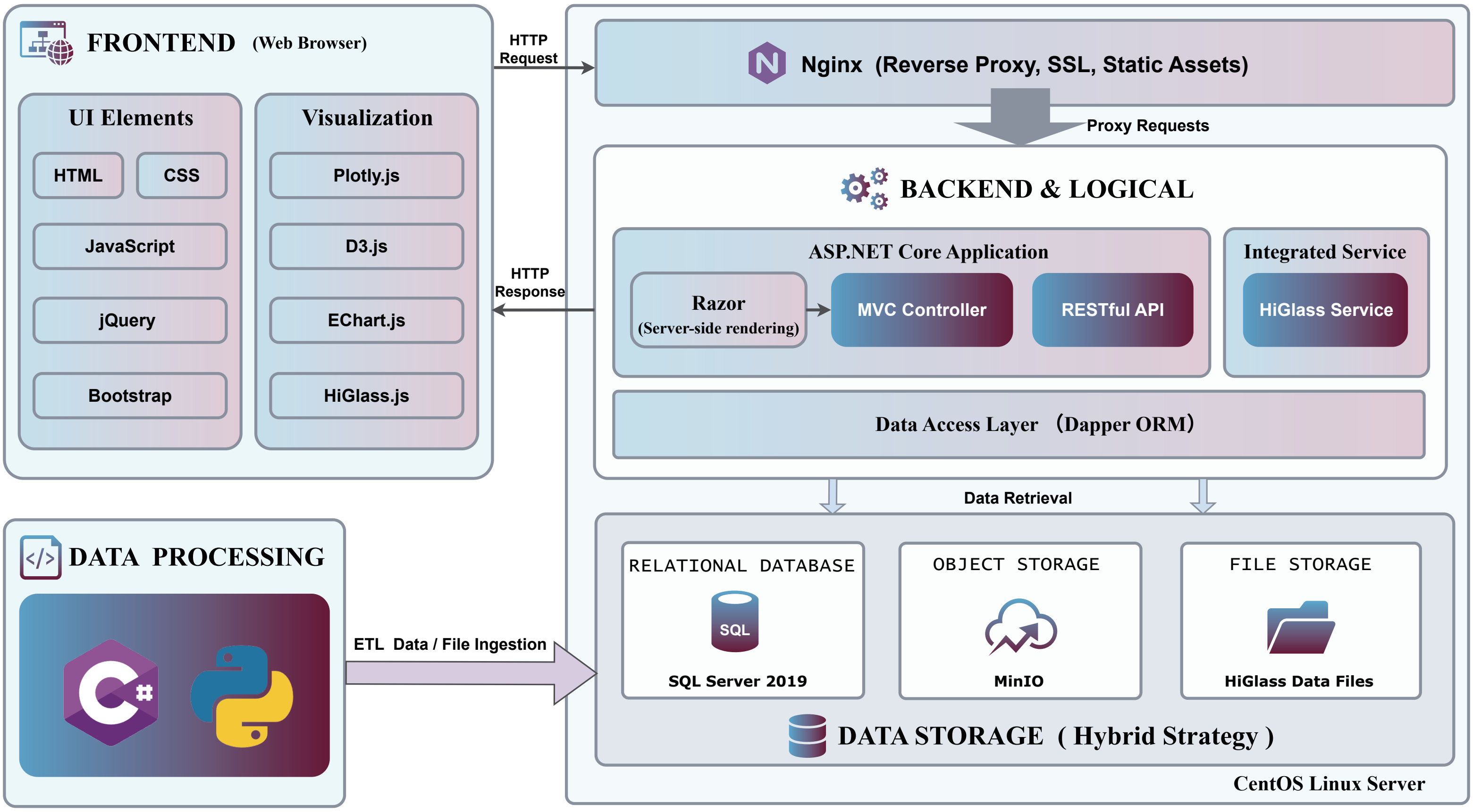

Figure S1: System architecture of Pig Matrix.
Pig Matrix adopts a four-tier client–server architecture consisting of a browser-based frontend, backend and logical services, an external data-processing layer, and a hybrid data-storage layer. Nginx serves as the web server and reverse proxy, routing browser requests to ASP.NET Core services, RESTful APIs, and the HiGlass service. The data-processing layer supports ETL processing and file ingestion, whereas SQL Server, MinIO object storage, and dedicated HiGlass data files together support structured annotation queries, large-scale file storage, and interactive 3D genome visualization.
